# Porous Zirconia Scaffolds Functionalized with Calcium Phosphate Layers and PLGA Nanoparticles Loaded with Hydrophobic Gentamicin

**DOI:** 10.3390/ijms24098400

**Published:** 2023-05-07

**Authors:** Iwona Pudełko, Anna Moskwik, Konrad Kwiecień, Sven Kriegseis, Małgorzata Krok-Borkowicz, Karolina Schickle, Dorota Ochońska, Piotr Dobrzyński, Monika Brzychczy-Włoch, Jesus Gonzalez-Julian, Elżbieta Pamuła

**Affiliations:** 1 Department of Biomaterials and Composites, Faculty of Materials Science and Ceramics, AGH University of Science and Technology, Al. Mickiewicza 30, 30-059 Kraków, Poland; 2 Department of Ceramics and Refractory Materials, Institute of Mineral Engineering, RWTH Aachen University, Forckenbeckstraße 33, 52074 Aachen, Germany; 3 Department of Restorative Dentistry and Endodontology, Justus-Liebig-University Giessen, Schlangenzahl 14, 35392 Gießen, Germany; 4 Department of Molecular Medical Microbiology, Chair of Microbiology, Faculty of Medicine, Jagiellonian University Medical College, 18 Czysta Str., 31-121 Kraków, Poland; 5 Centre of Polymer and Carbon Materials, Polish Academy of Sciences, 34 Curie-Sklodowskiej Str., 41-819 Zabrze, Poland

**Keywords:** zirconia scaffolds, porous scaffolds, implant-related infections, foam replication, polymer nanoparticles, PLGA, drug delivery, antimicrobial properties, hydrophobic gentamicin, AOT

## Abstract

Implant-related infections are a worldwide issue that is considered very challenging. Conventional therapies commonly end up failing; thus, new solutions are being investigated to overcome this problem. The in situ delivery of the drug at the implant site appears to be more sufficient compared to systemic antibiotic therapy. In this study, we manufactured porous zirconia scaffolds using the foam replication method. To improve their overall bioactivity, they were coated with a calcium phosphate (CaP) layer containing antibiotic-loaded degradable polymer nanoparticles (NPs) obtained by the double emulsion method to achieve the antibacterial effect additionally. Encapsulation efficiency (EE) and drug loading (DL) were superior and were equal to 99.9 ± 0.1% and 9.1 ± 0.1%, respectively. Scaffolds were analyzed with scanning electron microscopy, and their porosity was evaluated. The porosity of investigated samples was over 90% and resembled the microstructure of spongy bone. Furthermore, we investigated the cytocompatibility with osteoblast-like MG-63 cells and antimicrobial properties with *Staphylococcus aureus*. Scaffolds coated with a CaP layer were found non-toxic for MG-63 cells. Moreover, the presence of antibiotic-loaded nanoparticles had no significant influence on cell viability, and the obtained scaffolds inhibited bacteria growth. Provided processes of fabrication of highly porous zirconia scaffolds and surface functionalization allow minimizing the risk of implant-related infection.

## 1. Introduction

Bone is a connective tissue that supports the living body and protects all important organs, such as the brain, heart, or lungs [1]. In general, bone tissue exhibits the natural ability to regenerate, although self-healing capabilities are limited, and not all bone defects can be healed without surgical intervention [2,3,4]. A bone that cannot heal itself must be reconstructed or replaced [5,6]. Up to date, the golden standard for these kinds of defects is transplantation. However, there are different limitations, such as the lack of available tissue, donor site morbidity, or the risk of potentially fatal infections, that are associated with traditional auto- and allografting techniques [7]. To overcome these issues, reconstruction with artificial materials can be provided [1]. Synthetic porous scaffolds can mimic the properties of living bone, such as bioactivity, porosity, morphology, and strength [8].

According to statistical data, bone fractures remain a global challenge [9]. Approximately 6.2 million bone fractures occur each year, and around 500,000 bone graft surgeries are performed in the U.S. alone, making bone fractures one of the most common orthopedic interventions [8,10]. Moreover, the approximate value of sales of products related to tissue engineering in the U.S. in 2017 was equal to USD 9 billion [3]. On the other hand, in 2004, more than 100,000 implantations resulted in bone infections in U.S. only [11]. Implant-related infections, such as osteomyelitis, are a real burden [11,12]. They are difficult to treat and can cause several complications, such as inflammatory response [13] and bone loss needing complex revision procedures [12,14]. Furthermore, bacterial infections can lead to implant failure [12,14,15]. The current clinical treatment of implant-related infections includes systemic antibiotic therapy, but the rate of treatment failure is approximately 20%. Conventional therapy is not always effective due to the biological properties of bone tissue [12] and the formation of bacterial biofilms [15]. Typical antibiotic therapy is inefficient in the biofilm and gives unsatisfactory results [12]. For bone infection, local delivery of antibiotics with increased efficacy at the implant-tissue interface is a better solution to combat bacteria [12,14,15]. This approach provides lower but more targeted doses of antibiotics compared to traditional therapy [14]. As the local antibiotic carriers, the nano-, and microparticles (NPs and MPs, respectively) have been widely investigated. NPs, due to their size, are able to pass through biological barriers that MPs are unable to cross [16]. NPs may be fabricated from different materials; however, polymer ones are the most widely examined. Among polymers, natural such as chitosan and alginate, and synthetic such as polycaprolactone (PCL), poly(lactic acid) (PLA), or poly(lactic-*co*-glycolic acid) (PLGA) are used [16,17]. In addition, various antibiotics may be encapsulated in these particles, for example, gentamicin [18,19,20], vancomycin [21,22,23], or other biologically active substances [16,24,25]. Due to the hydrophilic nature of gentamicin, the encapsulation of this antibiotic in polymers, such as PLGA, may be challenging [3]. Furthermore, the hydrophilic character limits the capability of the drug to penetrate cells [26]. According to the literature, by changing the nature of gentamicin to a hydrophobic one, it is possible to achieve encapsulation efficiency and drug loading values in hydrophobic polymers higher than those obtained for gentamicin sulfate. It was reported that the hydrophobic ion-pairing of gentamicin sulfate with dioctyl sulfosuccinate sodium salt (AOT) can be applied to obtain a hydrophobic complex of gentamicin (gentAOT).

The demand for alternative therapies for bone tissue defect treatment involving scaffolds is growing due to the lack of tissue donors [2]. The artificial scaffolds perform several functions, such as filling the defect, supporting mechanical loading, and providing an environment suitable for cell growth and new bone formation. The scaffold should be bioactive and have appropriate mechanical properties [6,8]. In addition, the scaffold should exhibit an open porosity with a macropore size of 100–900 µm [7] since the proliferation and differentiation of osteoblasts are significantly influenced by the size of the pores [5,27]. Pore sizes larger than 300 µm are recommended to improve new bone formation through vascularization, while pore sizes in the range of 100–400 µm are suggested as appropriate for osteoconduction [28]. However, researchers imply that different sizes are favorable for bone regeneration [29].

The polymer foam replication method is one of the most well-known, simple, and economical techniques for the fabrication of porous scaffolds [8,10,30]. Different ceramic materials are being processed with this method, such as alumina [8], hydroxyapatite [7], porcelain [30], or zirconia [1,31]. Among mentioned materials, zirconia, i.e., zirconium oxide (ZrO_2_), is a widely used inorganic biomaterial that is considered a new bone reconstructive material [1]. Due to its properties, such as the highest for ceramic materials fracture toughness, corrosion resistance, and biocompatibility with bone tissue, it exhibits great potential for bone implant applications [15]. However, ZrO_2_ is characterized by low biological activity, contrary to calcium phosphates, which are the main components of the bone extracellular matrix [29]. The bioactivity of zirconia may be enhanced by surface modification [15]. Various methods are used to alter the surface of zirconia ceramics, such as sandblasting, base or acid treatments, and polishing. Moreover, improved bioactivity can also be achieved by functionalizing the surface with bioactive materials [15,32].

In this study, we hypothesized that the CaP-based bioactive layer with antibiotic-loaded NPs deposited on ZrO_2_ substrates merges good mechanical performance with efficient bioactivity and bactericidal effects. It was previously proven that coatings with the ability to release drugs reduce infections associated with implants in both short- and long-term studies [33]. In this work, zirconia scaffolds were fabricated using the foam replication method, and the CaP layer was deposited by the biomimetic co-precipitation method. We investigated the microstructure and porosity of obtained scaffolds, likewise the morphology of the coating. Instead of gentamicin sulfate (gentS), hydrophobic gentamicin was synthesized and encapsulated in PLGA to achieve better drug loading capacity, as previously reported [3,26,34,35]. To obtain NPs, we used solid-in-oil-in-water (S/O/W) emulsification. The encapsulation efficiency, drug loading, morphology, and size distribution were examined, and the drug release profiles were investigated. Biological evaluation was performed with osteoblast-like MG-63 cells. Antimicrobial properties were verified with *Staphylococcus aureus.* We assume that a zirconia scaffold coated with a bioactive layer capable of releasing the antibiotic will provide a superior solution for alternative therapies for bone tissue treatment.

## 2. Results

### 2.1. Morphology, Size Distribution, and Encapsulation Efficiency of NPs

**Figure 1:**
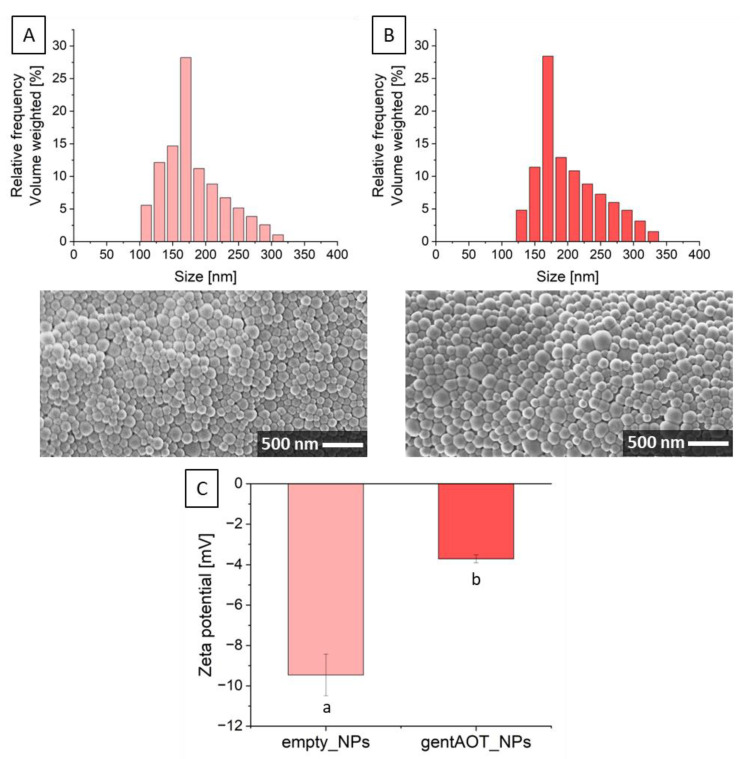
Size distribution and SEM pictures of empty_NPs (**A**) and gentAOT_NPs (**B**) and Zeta potential values (**C**) for empty_NPs and gentAOT_NPs. Different lowercase letters mean statistically significant difference (*p* < 0.05).

The morphology of the obtained particles was evaluated by applying SEM. Both empty (Figure 1A) and loaded NPs (Figure 1B) were of spherical shape and nanometric, homogeneously distributed size. Furthermore, the surface of the investigated particles was smooth. The size analysis with the particle analyzer confirmed the nanometric size of the NPs. For empty NPs (empty_NPs), the average size was equal to 214.6 ± 14.0 nm, while for gentAOT-loaded NPs (gentAOT_NPs), it was 236.7 ± 33.6 nm. The size distribution (Figure 1) was similar in both cases. Empty NPs size was between 100 and 320 nm, while the size range for loaded particles was 120–340 nm. The size distribution of gentAOT_NPs was slightly shifted to the highest values, and that is why bigger particles can be observed in Figure 1B. Although, the highest peak for both types of NPs particles was located in the same position. Moreover, no statistically significant difference in the size of both NPs types was found, which means that they belong to the same population. The presence of gentAOT had no impact on the size of particles. The Zeta potential (Figure 1C) for empty NPs was equal to −9.5 ± 1.0 mV, while for gentAOT-loaded NPs, the value of electrokinetic potential significantly increased and was −3.7 ± 0.2. That indicates that the presence of gentAOT influences the surface potential of particles.

The encapsulation efficiency and drug loading of obtained nanoparticles were checked with the use of reaction with OPA reagent and measurement of fluorescence. Table 1 presents different parameters of fabricated NPs. Results of EE show that hydrophobic gentamicin may be easily encapsulated in PLGA. The encapsulation process is superior, and the effectiveness is almost 100%. Moreover, the gentAOT accounts for over 9% of the weight of fabricated particles.

### 2.2. Characterisation of Porous Scaffolds

**Figure 2:**
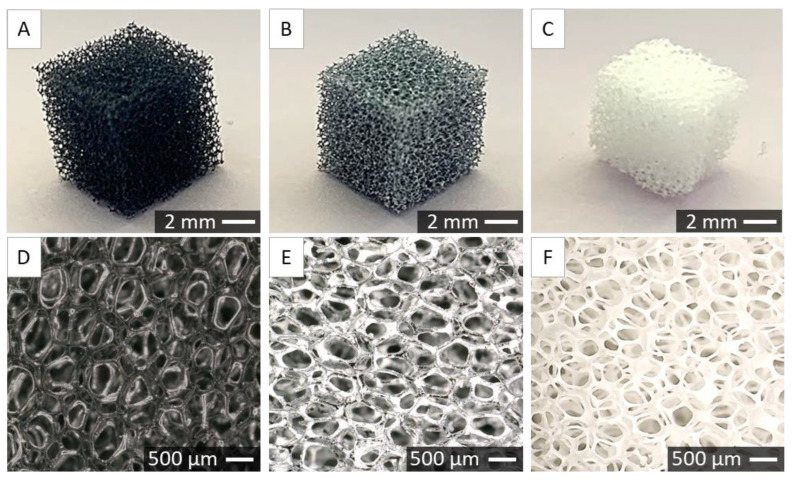
Gross morphology (**A**–**C**) and optical microphotographs (**D**–**F**) of PU sponge (**A**,**D**), sponge covered with zirconia ceramic slurry (**B**,**E**), and sintered zirconia scaffold (**C**,**F**).

Scaffolds were fabricated using the foam replication method. In Figure 2 the gross morphology of polyurethane (PU) foam (Figure 2A), foam covered with ceramic slurry (Figure 2B), and sintered ceramic scaffold (Figure 2C) are shown. In Figure 2D–F, corresponding microstructures observed under optical digital microscope are shown. One can observe that after sintering, the struts are thinner, and the pores are bigger than in the initial polyurethane foam.

**Figure 3:**
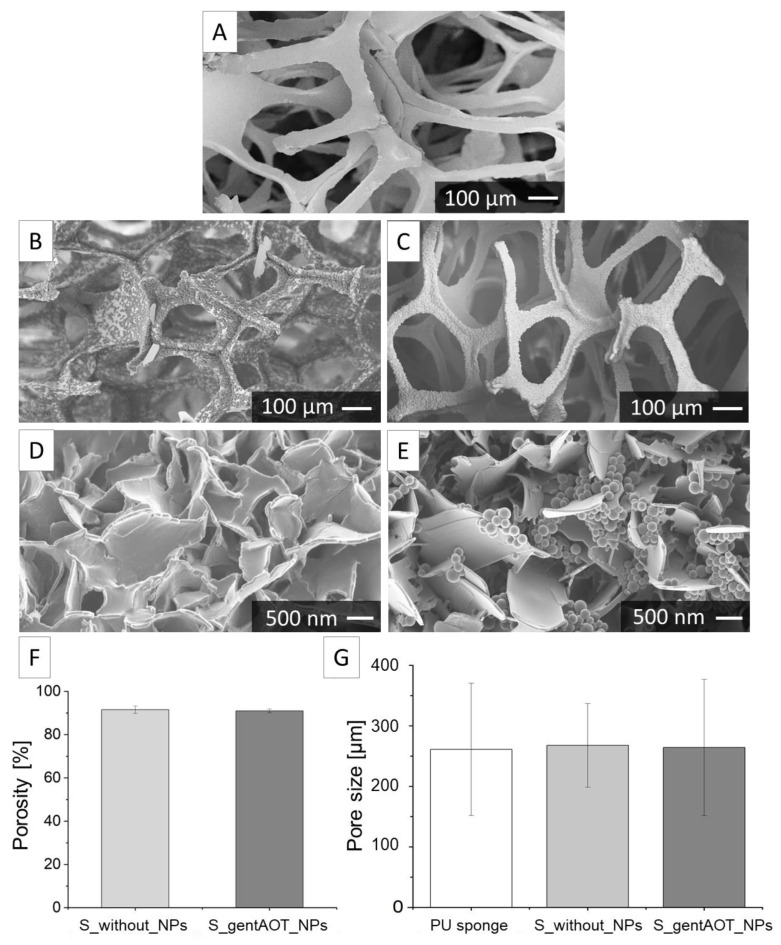
SEM images (**A**–**E**), porosity (**F**), and pore size (**G**) of scaffolds. (**A**)—scaffold after sintering; (**B**)—scaffold after the first step of coating with CaP; scaffold after the second step of coating with CaP (**C**–**E**). Picture (**E**) shows the final CaP coating with introduced gentAOT_NPs.

SEM images present the microstructure of an intact ZrO_2_ sintered scaffold (Figure 3A). Subsequently, zirconia scaffolds were coated with CaP in a two-step precipitation process. There was a visible difference between the CaP crystals obtained after only one step of the coating process (Figure 3B) compared to those after the second step (Figure 3C,D). The surface of the scaffold after only the first step of precipitation was not homogenously coated, whereas after the second step of precipitation, the CaP-crystals created a homogenous layer on the zirconia scaffold. The impact of the presence of nanoparticles in the coating process was investigated (Figure 3E). The CaP crystals visible in Figure 3E did not differ from those presented in Figure 3D, indicating that the CaP precipitation was not affected by the presence of NPs in the solution during the coating process. Moreover, we can observe that the attached NPs to the CaP crystals are randomly distributed on the scaffold surface within the CaP coating.

The porosity of sintered scaffolds was examined by hydrostatic method. The results (Figure 3F) show that the average porosity of the scaffolds without particles (S_without_NPs) was 91.5 ± 1.5%, while for the scaffolds containing gentAOT_NPs (S_gentAOT_NPs) it was equal to 91.0 ± 0.8%. According to statistical analysis, there was no significant difference between both scaffold groups. The pore size varied from 191 µm to 422 µm for sintered scaffold and from around 87 µm to 713 µm for the scaffold coated with a CaP layer containing gentAOT-loaded NPs. Although, the average values exhibited no statistically significant differences (Figure 3G).

### 2.3. Drug Release 

**Figure 4:**
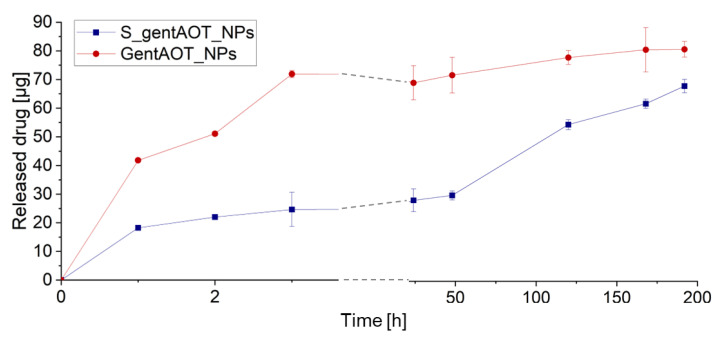
Drug release profiles of scaffolds containing gentAOT-loaded nanoparticles and of NPs themselves during incubation in PBS.

To investigate drug release, we incubated gentAOT_NPs and S_gentAOT_NPs in PBS under dynamic conditions at 37 °C. In predetermined periods, we collected 2 mL of solutions and replaced them with 2 mL of fresh PBS; the concentration of released gentamicin was measured. There was a significant difference between the concentration of gentamicin released from the scaffold compared to the free NPs (Figure 4). The release of antibiotic was faster, and there was more drug in the solution in the case of gentAOT_NPs. The concentration of gentamicin was lower for S_gentAOT_NPs. In the beginning, the drug was released in a similar manner for both types of investigated samples; however, after 3 h, the concentration stabilized in the case of gentAOT_NPs, while for S_gentAOT_NPs it was observed a continuous increase in released drug concentration.

### 2.4. Antimicrobial Efficiency

**Figure 5:**
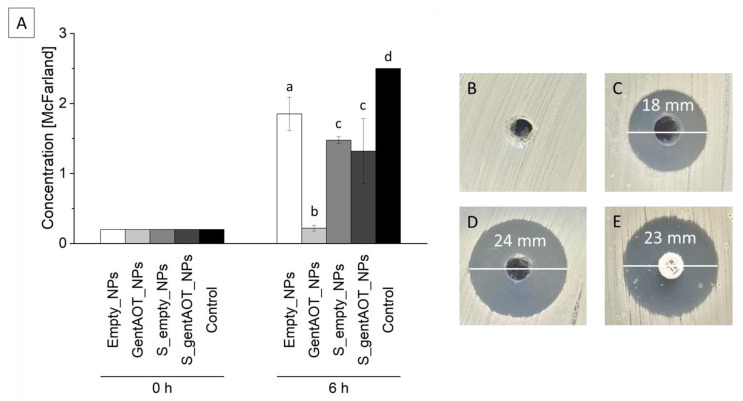
Concentration of bacteria after 6 h culture in the presence of extracts of different samples (**A**); time 0 h represents the background, which is the optical density of the tubes and not the concentration of bacteria. Different lowercase letters mean statistically significant difference (*p* < 0.05). Representative bacteria growth inhibition zones: (**B**)—Empty_NPs; (**C**)—GentAOT_NPs (500 µg/mL); (**D**)—GentAOT_NPs (2000 µg/mL); (**E**)—reference disc (10 µg of gentamicin).

We performed tests with *S. aureus* in order to investigate the antibacterial effect of our scaffolds. Extracts of the scaffolds containing empty or loaded nanoparticles (S_empty_NPs or S_gentAOT_NPs, respectively) and the nanoparticles themselves were added to the bacteria suspension and cultured for 6 h. All investigated samples decreased the bacterial growth rate compared to the control (Figure 5A). The best result we observed for gentAOT_NPs, which significantly reduced the number of bacteria in the suspension. The concentration of bacteria, in this case, had barely changed and was equal to 0.22 ± 0.04 McFarland, while the initial value was 0.2. This means that the amount of antibiotic released from the NPs within the first 6 h is enough to inhibit bacterial growth. There was no statistically significant difference between S_empty_NPs and S_gentAOT_NPs. This suggests that the number of gentAOT-loaded NPs immobilized on the scaffold is rather low. Nevertheless, the bacterial growth rate for S_gentAOT_NPs was much lower compared to control. Interestingly, the presence of extract from S_empty_NPs inhibited bacterial growth slightly better than the extract of empty particles. 

Since we observed the best results for gentAOT_NPs, we performed the Kirby–Bauer test with the use of suspension of particles in PBS to confirm their antibacterial properties. As a control, we used a disc containing 10 µg of gentamicin and empty nanoparticles. The results (Figure 5) show that both of the drug-loaded examined samples inhibited bacterial growth on agar plates. The diameter of the inhibition zone for a more concentrated suspension was naturally larger since a higher amount of antibiotic could be released. However, the suspension of concentration of 2000 µg/mL (Figure 5D) exhibited an even bigger inhibition zone than the reference disc (Figure 5E), which confirmed the antibacterial properties of the nanoparticles loaded with gentAOT. 

### 2.5. Biological Evaluation

**Figure 6:**
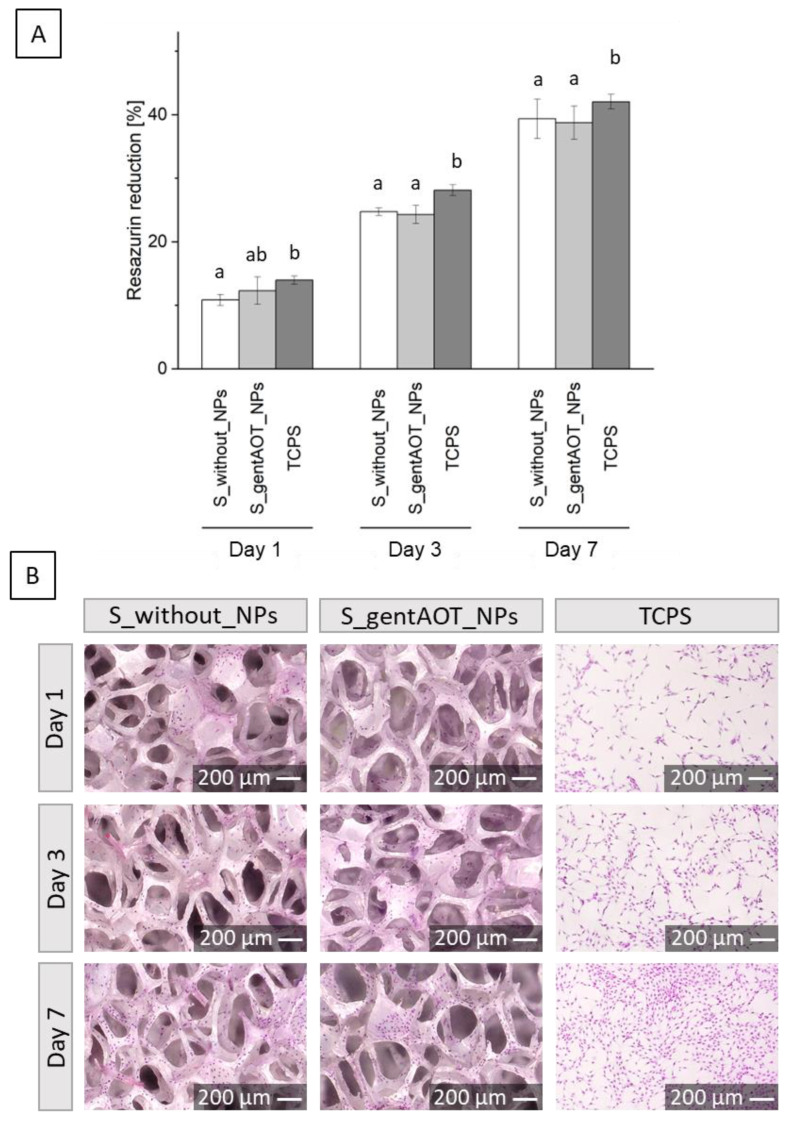
MC3T3 cell metabolic activity (**A**) and cell distribution after hematoxylin-eosin staining (**B**) on scaffolds covered with CaP layer without NPs (S_without_NPs) and containing gentAOT-loaded nanoparticles (S_gentAOT_NPs) and on TCPS. Different lowercase letters mean statistically significant difference (*p* < 0.05).

We examined the cellular response of fabricated scaffolds with osteoblast-like MG-63 cells to confirm their cytocompatibility. Briefly, cells were seeded in the samples and on tissue culture polystyrene (TCPS) as a control and cultured for 1, 3, and 7 days. According to the AlamarBlue results (Figure 6A), cells proliferated well on all surfaces. There were no statistically significant differences between scaffolds containing gentAOT_NPs compared to scaffolds without particles after each time point of the study. In comparison to S_gentAOT_NPs, the proliferation was slightly higher on TCPS, although after the first day, there was no statistically significant difference. In S_without_NPs, cell proliferation was lower than in TCPS. However, the resazurin reduction increased for each sample in the same manner and was approximately 40% after 7 days of culture. In addition, we stained cells with hematoxylin and eosin and observed their morphology, distribution, and proliferation with an optical digital microscope (Figure 6B). Cells were well spread on the surface, and the number of cells increased with culture time.

## 3. Discussion

Polymers are materials that are widely investigated for different biomedical applications. Especially PLGA, as a material approved by the Food and Drug Administration (FDA) and the European Medicines Agency (EMA), is often used in medical devices such as degradable sutures and screws for osteosynthesis and osteochondral fixation [19,36,37]. Nanoparticles fabricated with the use of PLGA, loaded with drugs or different active substances, are of great interest. The size of nanoparticles is especially important in bone tissue because nanosized particles are able to penetrate the bone structure [38,39]. Researchers report different sizes of NPs that range from 100 to 600 nm. Many studies present particles with a size of about 200 nm [19,26,37,40]. The size of the particles obtained in our work is 214 ± 14 nm and 237 ± 34 nm for empty and gentAOT-loaded particles, respectively. Apart from the size of NPs, encapsulation efficiency, and drug loading are crucial factors that determine the utility of polymer nanoparticles as drug carriers. In the literature, there are numerous reports that present the encapsulation of gentamicin sulfate. EE and DL values reported are in the range of 2–50% and 0.5–6%, respectively. These numbers change depending on the conditions of fabrication, materials, and the size of particles. Posadowska et al. obtained several types of PLGA nanoparticles by changing manufacturing parameters, such as gentamicin concentration in the oil phase and poly(vinyl alcohol) (PVA) concentration in the water phase. In conditions similar to ours, they received particles with a size of 219 ± 11 nm and the EE and DL equal to 11.2 ± 1.5% and 0.34 ± 0.02%, respectively [40]. Abdelghany et al. reported different particle sizes (240–360 nm) with DL up to 22.4 ± 2.5 µg per 1 mg of PLGA [41]. Although the size of the nanoparticles is rather easy to control and is mostly satisfactory, the EE and DL may be improved. Modification of gentamicin sulfate into hydrophobic gentamicin allows for significantly higher values of EE and DL. According to the literature, the efficiency of gentAOT encapsulation varied from 90 to 100%, and DL of even 60 µg of gentamicin per 1 mg of particles was reached [26]. The encapsulation efficiency and drug loading of our nanoparticles are equal to 99.9 ± 0.1% and 9.1 ± 0.1%, respectively. The superior EE and DL, especially compared to gentamicin sulfate, make hydrophobic gentamicin an interesting solution to be encapsulated in hydrophobic PLGA. 

The foam replication method was selected as an effective technique for obtaining porous zirconia scaffolds. Adjusted sintering conditions allowed us to fabricate samples without surface defects. Although zirconia is a biocompatible material with bone exhibiting good mechanical properties, its cellular response may be improved. Thus, a biomimetic co-precipitation process was performed in order to obtain a CaP layer on the surface of sintered scaffolds. Nanoparticles were introduced to the second step of the coating process to immobilize them to the CaP crystals. The applied method allowed us successfully attach NPs to the CaP layer. Obtained CaP crystals are similar to those previously reported [42,43]. According to SEM images, nanoparticles were rather homogenously distributed between crystals and had no negative impact on the CaP precipitation process. The biomimetic co-precipitation process that we used to functionalize zirconia scaffolds was reported previously [44]. According to our former results, the thickness of the coating was equal to 1.6 ± 1.2 µm and 3.9 ± 1.2 µm after the first and the second step of the coating process, respectively. The addition of NPs did not influence the thickness of the CaP layer. The X-ray diffraction results confirmed the presence of apatitic phases after both steps of the coating process [44].

To provide a large surface area that is necessary for cell interaction and space for extracellular matrix creation, a highly porous scaffold with a porosity of at least 90% is required [29,45]. The porosity of our scaffolds with or without particles was equal to 91.5 ± 1.5% and 91.0 ± 0.8%, respectively. There were no statistically significant differences between both values, which means that the addition of particles did not influence the porosity. Moreover, according to the literature, the high scaffold porosity is appropriate for enhanced interfacial reactions with cells. According to the literature, different pore sizes are required for bone regeneration [27]. Scaffolds obtained in our investigation are characterized by an average pore size of 260 µm for coated and uncoated scaffolds. This size is within the range considered to be suitable for bone formation [28]. 

Drug release from gentAOT_NPs and S_gentAOT_NPs was investigated. This knowledge is crucial to determine the antibacterial effect against *S. aureus* without provoking cytotoxic responses of cells. Nevertheless, at first, the antibiotic was released from both the scaffold and the particles themselves in a similar manner. After a while, the concentration started stabilizing in the case of gentAOT_NPs, whereas, for S_gentAOT_NPs, it was continuously increasing but was still significantly lower as compared to the nanoparticles themselves. The release profile suggested that after a burst release, the drug was released slowly and in small portions. Analyzing the differences between the concentration of drug released from S_gentAOT_NPs, and gentAOT_NPs, we can conclude that the number of particles immobilized on the surface of the scaffolds was lower than the initial amount introduced into the coating process. At the same time, the release itself was slower. This may suggest that the particles present in the scaffold were less exposed to the solution, resulting in slower but prolonged and more sustainable drug delivery in comparison to gentAOT-loaded nanoparticles. 

According to Boo et al., despite the superior EE, DL, and antibacterial properties, the toxicity of gentAOT should be considered [35]. The biological evaluation showed that osteoblast-like MG-63 cells proliferated well in both samples. The presence of gentAOT_NPs had no negative impact on the viability of cells compared to the scaffold without particles. This means that the concentration of gentAOT was non-toxic for MG-63 cells. At the same time, the amount of antibiotic released from the scaffolds should be improved to achieve an appropriate antibacterial effect. The antimicrobial activity of gentAOT against different strains of bacteria has already been reported [34,35]. In our study, the bacterial growth was highly decreased in the presence of gentAOT_NPs, while for scaffolds that contained antibiotic-loaded particles, the growth inhibition was not significantly higher as compared to the scaffolds with empty_NPs. However, as mentioned above, there are fewer particles on the surface than the initial amount used in the coating process. There is a need to improve the immobilization process of the particles in the CaP layer to increase the number of particles attached to it. Furthermore, the toxicity of gentAOT should be taken into account. 

Moreover, the Kirby–Bauer test revealed great antibacterial properties of gentAOT_NPs suspended in water. The inhibition zones for concentrations 500 µg/mL and 2000 µg/mL were equal to 18 and 24 mm, while for the reference disc, it was equal to 23 mm. That means that more than 10 µg of antibiotic was released from the particles within the duration of an assay, which is, in fact, 4 h of diffusion during the pre-incubation time. Although the antimicrobial properties of the investigated suspensions were remarkable, biological evaluation with different types of cells is required to confirm the cytocompatibility of examined materials. The concentration of gentAOT-loaded particles of 1000 µg/mL was reported to be toxic for MC3T3 cells, while the concentration of 500 µg/mL was safe for investigated cells; however, the amount of antibiotic encapsulated in the used NPs was higher [3]. This indicates that we achieved an antibacterial effect in a safe concentration. At the same time, too high concentration of unmodified gentamicin sulfate has also been reported to be toxic [46]. That is why the number of particles and the drug loading have to be investigated more deeply. Nevertheless, the use of gentAOT gives a better antibacterial effect, with higher values of encapsulation efficiency/drug loading and great repeatability. 

To the best of our knowledge, there is still a gap in the literature on studies of hydrophobized antibiotics, such as hydrophobic gentamicin. Although, it was reported that the antimicrobial properties of such drugs are superior compared to non-modified antibiotics. Moreover, the simplicity of this conversion combined with the remarkable EE and DL may allow us to obtain drug release systems whose properties are easy to control. Taking into account the difficulties associated with implant-related bone infections, the solution we propose may be an effective way to treat these diseases. 

## 4. Materials and Methods

### 4.1. Materials

PLGA (Mw = 99,800 g/mol, polydispersity index = 1.8) was synthesized by ring-opening polymerization with the zirconium(IV) acetylacetonate Zr(acac)_4_ initiator in the Polish Academy of Sciences, Zabrze, Poland, with a lactide to glycolide ratio equal to 85:15, according to a method described previously [47]. Gentamicin sulfate, dioctyl sulfosuccinate sodium salt, poly(vinyl alcohol) (PVA, Mw = 31,000 g/mol, Mowiol 4–88), o-phtaldialdehyde, 2-merkaptoethanol, calcein-AM, propidium iodide, resazurin were provided from Sigma Aldrich, Steinheim, Germany. Chemicals used for biomimetic coatings, such as sodium chloride (NaCl), potassium chloride (KCl), and magnesium chloride hexahydrate (MgCl_2_ · 6H_2_O), came from Th. Geyer GmbH & Co. KG, Hamburg, Germany, and calcium chloride dihydrate (CaCl_2_ · 2H_2_O), sodium dihydrogen phosphate dihydrate (NaH_2_PO_4_ · 2H_2_O), sodium bicarbonate (NaHCO_3_), potassium hydrogen phosphate (K_2_HPO_4_) were provided by Merck KGaA, Darmstadt, Germany. Dichloromethane (DCM), methanol, and ethanol came from Chemland, Stargard, Poland. Glycerine, sodium hydroxide (NaOH) and phosphoric acid (H_3_PO_4_), acetic acid, and sodium acetate were provided by POCH, Gliwice, Poland. Polyurethane foams were purchased from Caligen Europe B.V., BC Breda, The Netherlands. The zirconium dioxide powder stabilized with 3-mol-yttrium (3-mol-Y_2_O_3_) (YSZ) was obtained from Tosch Corporation, Yamaguchi, Japan. Dolapix CE64 (CE64) was provided by Zschimmer und Schwarz, Lahnstein, Germany. Modified Eagle Medium (MEM), fetal bovine serum (FBS), and mixture of penicillin and streptomycin for cell culture were purchased from PAN Biotech, Aidenbach, Germany. Phosphate-buffered saline (PBS) came from VWR Life Science, Radnor, PA, USA.

### 4.2. Manufacturing of Nanoparticles Loaded with Hydrophobic Gentamicin

To prepare gentamicin-loaded NPs, the double emulsion method with solvent evaporation was used. However, we modified gentamicin sulfate by converting it into hydrophobic gentamicin, as previously described [3], and used it instead. In the first step of gentAOT_NPs fabrication, 6 mg of gentAOT was added to 3 mL of a 2% solution of PLGA in DCM and mixed with ultrasounds for 3 min with an amplitude of 40% (Vibra Cell VCX130, Sonics, Newtown, CT, USA). Then, we slowly added the antibiotic-containing PLGA solution to the 2% aqueous solution of PVA and mixed it with ultrasounds on ice for 3 min with an amplitude of 40% and then on a magnetic stirrer with a speed of 1000 rpm for 24 h. Subsequently, the solutions were centrifuged (MPW-351R, MPW Med. instruments, Warszawa, Poland) once for 20 min at a speed of 15,000 rpm at 4 °C. We removed the supernatants and kept 2 mL to investigate the encapsulation efficiency and drug loading. Then, we added 20 mL of ultra-high quality water (UHQ water, produced in Direct-Q 3UV, Merck Millipore, Burlington, VT, US) and centrifuged the particles again. This process was repeated 3 times. After removing the water from the last centrifugation, we added 5 mL of fresh UHQ water and kept it at the temperature of −80 °C for 24 h. The last step in the preparation of particles was the freeze-drying process, which was carried out for 24 h (Christ Alpha 1–2 LDplus, Martin Christ, Osterode am Harz, Germany). To prepare empty_NPs, we omitted mixing antibiotics with PLGA in the first step, and PLGA was sonicated alone to keep the same conditions. The rest of the process was conducted without any changes. The particles were stored at −20 °C prior to further use.

### 4.3. Nanoparticles Characterization

The shape and size of the NPs were observed by scanning electron microscopy (SEM, GeminiSEM 500, Zeiss, Oberkochen, Germany). To do so, they were attached to carbon tape and covered with a thin carbon layer. To confirm the size of NPs and check the size distribution, we used the particle analyzer (Litesizer 500, Anton Paar, Graz, Austria). Briefly, we suspended NPs in UHQ water and transferred them to a disposable sizing cuvette to measure their size. Additionally, the zeta potential of obtained NPs was measured. Particles were prepared in the same way as for the size measurements, and the suspension was placed in a cuvette to measure the surface zeta potential (Zetasizer nano-ZS, Malvern, UK).

The EE and DL were investigated with the use of supernatants kept after the first centrifugation of NPs. To quantify the amount of drug in the solution, the OPA assay was conducted. This test is based on the reaction of o-phtaldialdehyde with gentamicin present in the supernatant and the fluorescence measurement. To do so, the OPA reagent was prepared by mixing o-phtaldialdehyde with methanol, 2-mercaptoethanol, and borate buffer. The supernatant (three times for 50 µL) was transferred to a black 96-well plate, and 50 µL of OPA reagent was added to each well-containing samples. Then, we measured fluorescence (λ_ex_ = 340 nm, λ_em_ = 460 nm, FLUOstar Omega, BMG Labtech, Ortenberg, Germany) and quantified EE and DL according to Formulas (1) and (2), respectively [3,44].

(1) $$EE=\frac{mass\;of\;used\;drug-mass\;of\;drug\;in\;supernatant}{mass\;of\;used\;drug}\times 100\%$$

(2) $$DL=\frac{mass\;of\;used\;drug-mass\;of\;drug\;in\;supernatant}{mass\;of\;obtained\;NPs}\times 100\%$$

### 4.4. Zirconia Suspension Preparation

Zirconium dioxide stabilized with 3 mol of yttrium (3-mol-Y_2_O_3_) (YSZ) powder was used to prepare the ceramic slurry. First, the basic suspension was manufactured. In brief, YSZ was mixed with distilled water and CE64 in the following ratio (volume percentage): 58.55% of water, 1.75% of CE64, and 39.70% of YSZ. The suspension was ground in an agitator mill (MicroCer, Netsch-Feinmahltechnik GmbH, Selb, Germany) for 12 h at a speed of 2500 rpm. During this process, we used zirconia grinding beads with a diameter of 0.2–0.3 mm (Zeta Beads, Netzsch-Feinmahltechnik GmbH, Selb, Germany). The basic suspension was then mixed with additives, as shown in Table 2. After that, the slurry was ready to use. The suspension was stored in a low-density polyethylene (LDPE) bottle on a roller bench.

### 4.5. Porous Scaffold Fabrication

We obtained porous zirconia scaffolds using the foam replication method. To do so, we used PU sponges in a cube shape (1 × 1 × 1 cm^3^). They were etched in NaOH for 2 h at 37 °C and rinsed with UHQ water. Then, we covered sponges with ceramic slurry prepared as described in Section 4. This process includes 3 steps dipping foams into a suspension and removing excess slurry by squeezing and blowing with compressed air, followed by drying at 37 °C. During the first step, drying lasted 4 h, while in the second and third steps, it lasted 24 h. After that, sponges covered with a slurry were thermally treated. First, the PU foams needed to be buried out in the air for 30 min at 200 °C and then for 2 h at 600 °C, and subsequently, samples were sintered at 1450 °C for 2 h. 

### 4.6. Precipitation of CaP

The sintered scaffolds were treated with 5 M phosphoric acid (H_3_PO_4_). Briefly, we placed scaffolds in a beaker filled with acid and kept them at 37 °C for 3 days, rinsed them with UHQ water, and dried them. To coat the scaffolds with a CaP layer, the co-precipitation method with the use of solutions similar to concentrated simulated body fluid (SBF) was applied, as we reported previously [44]. First, the scaffolds were placed in tubes which we filled with 15 mL of the solution prepared as previously described and left for 24 h at 37 °C [42]. Costa et al. presented a coating method in which they used different solutions for the first and second steps [43]. After we removed the solution from the tubes, we replaced it with 2.5 mL of solution for the second step as described by Costa et al. and added 5 mg of empty or gentAOT-loaded NPs to each tube (S_empty_NPs or S_gentAOT_NPs, respectively). During this step, the tubes with samples were shaken at a speed of 150 rpm at room temperature for 24 h. Subsequently, the scaffolds were rinsed three times by filling the tube with 15 mL of UHQ water and dried at 37 °C.

### 4.7. Scaffolds Characterization

With the use of an optical microscope, we observed the surface of the PU sponge, a sponge covered with ceramic slurry and sintered scaffolds. To check the quality of scaffolds and the effectiveness of the coating process as well as measure the size of pores, the SEM was used. We observed our samples after each step of preparation. In addition, the porosity was investigated for both coated and uncoated samples by the hydrostatic method. 

### 4.8. Biological Evaluation

We evaluated the cellular response with osteoblast-like cells MG-63 (European Collection of Cell Cultures, Salisbury, UK). The coated scaffolds (with or without NPs) were placed in a 24-well plate and sterilized in 70% ethanol for 15 min, followed by UV irradiation. On each scaffold, we seeded 10,000 cells suspended in 2 cm^3^ of minimal essential medium (MEM) supplemented with fetal bovine serum (FBS, 10%), antibiotics (mixture of penicillin and streptomycin, 1%), amino acids (0.1%) and sodium pyruvate (0.1%). Cell culture was carried out at 37 °C in a 5.0% CO_2_ atmosphere for 1, 3, and 7 days. The Alamar Blue assay was used to evaluate the metabolic activity of the cell. In brief, 2 mL of a 10% Alamar Blue solution in the medium was added to each well and incubated for 4 h. After this time, 100 µL of the solution was collected from each well, transferred to a black 96-well plate, and the fluorescence was measured (λ_ex_ = 544 and λ_em_ = 590 nm, FluoStar Omega, BMG Labtech, Ortenberg, Germany). We calculated the percentage of resazurin reduction using the Formula (3) [44]:

(3) $$\%rezasurin\;reduction=\frac{S_{x}-S_{blank}}{S_{reducted}-S_{blank}}\times 100\%$$

where:

S_x_—fluorescence of the samples,
S_blank_—fluorescence of MEM with 10% AlamarBlue reagent, without cells (0% reduction of resazurin),
S_reduced_—fluorescence of MEM with 10% AlamarBlue reagent autoclaved at 121 °C for 15 min (100% reduction of resazurin).

To investigate the morphology, distribution, and proliferation of cells, we stained them with hematoxylin and eosin and observed them with an optical microscope (VHX-900F, Keyence, Osaka, Japan). Briefly, samples were rinsed with PBS, and 2 mL of hematoxylin was added to the wells. After 5 min of incubation, the dye was removed from the wells, and the samples were rinsed with UHQ water until a clear solution. The process was repeated with eosin. After that, the samples were left for 24 h to dry and then observed with a microscope. 

### 4.9. Drug Release Profiles

Drug release profiles were explored to check the potential for the use of our scaffolds as drug delivery systems. We closed the scaffolds with NPs or 5 mg of nanoparticles themselves in dialysis bags with 2 mL of PBS and placed them in sealed vials filled with 20 mL of PBS. The experiment was carried out at 37 °C under dynamic conditions (shaking at a speed of 150 rpm). In predetermined periods of time, we collected 2 mL of PBS, which we replaced with the same amount of fresh PBS. Then, the amount of drug in the collected solution was quantified using the OPA assay, as described in Section 4.3. 

### 4.10. Antimicrobial Effect

To check the antibacterial properties of our samples, the tests with *Staphylococcus aureus* (ATCC MSSA 25923) were performed. We obtained extracts of scaffolds containing empty or loaded nanoparticles and from particles themselves as control. Briefly, samples were incubated in 2 mL of PBS for 24 h at 37 °C. To prepare extracts from the scaffolds, they were first pre-wetted with PBS to remove air from pores, as described previously [48]. The suspension of bacteria of a concentration of 0.5 McFarland was prepared. Extracts after incubation were filtered with 0.2 µm filters. Then, 200 µL of extracts were added to 1800 µL of 100-fold dissolved bacteria suspension (10% *v*/*v*) and cultured for 6 h at 37 °C. After that time, the concentration of bacteria in each sample was measured with a densitometer. In addition, to prove the antibacterial effect of gentAOT-loaded nanoparticles, we performed the Kirby–Bauer test with NPs. Suspensions of NPs in PBS of concentration of 2000 µg/mL and 500 µg/mL were prepared, and the 0.5 McFarland bacteria suspension was applied on agar plates. We cut out holes of size of 6 mm in agar with a sterile pipette tip and added 100 µL of adequate suspension. As reference disc containing 10 µg of gentamicin was used. Samples were pre-incubated for 4 h at 4 °C and then incubated for 24 h at 37 °C. Later on, growth inhibition zones were measured.

### 4.11. Statistical Analysis

Results are presented as average ± standard deviation (SD). Statistical analysis of results was performed using a one-way analysis of variance (one-way ANOVA) followed by the post-hoc LSD Fisher test in the OriginLab software, and the Shapiro–Wilk test was used to verify the normal distribution. As statistically significant, we considered when p* < 0.05.

## 5. Conclusions

The aim of this work was to functionalize the surface of zirconia ceramics to improve its cytocompatibility with model bone cells and to provide it with antimicrobial properties. Porous zirconia scaffolds were obtained using the foam replication method and covered with a calcium phosphate layer to improve bioactivity. Moreover, antibiotic-loaded degradable PLGA nanoparticles were immobilized in the coating to achieve controlled drug release and antibacterial effect. 

Hydrophobic gentamicin was encapsulated in PLGA nanoparticles. The size, Zeta potential, encapsulation efficiency, and drug loading of the nanoparticles were investigated. The conversion of gentamicin sulfate into hydrophobic gentamicin by ion pairing was found to be an effective way to fabricate particles with significantly improved encapsulation efficiency and drug loading values without changing the size of NPs; however, the Zeta potential was significantly increased. The scaffold preparation process, as well as the biomimetic co-precipitation, allowed us to obtain porous zirconia scaffolds with porosity greater than 90% and a pore size of approximately 260 µm. All types of samples were cytocompatible with osteoblast-like MG-63 cells. The presence of gentAOT-loaded nanoparticles had no negative impact on either the calcium phosphate layer deposition process or cell proliferation. Antimicrobial properties were satisfactory for gentAOT-loaded nanoparticles, while for the scaffold containing these particles, the antibacterial effect was lower, which can be correlated with lower drug doses released during extract preparation. 

On the one hand, we found that the antibacterial properties of nanoparticles containing hydrophobic gentamicin are superior. On the other hand, the toxicity of gentAOT has to be taken into consideration. All of this leads to the conclusion that more extensive research is necessary for modified gentamicin. Nevertheless, the whole process of manufacturing functionalized scaffolds is an effective technique to improve the bioactivity of the zirconia surface and achieve the antibacterial effect of the resulting scaffolds.

## Figures and Tables

**Table 1:** Characteristics of obtained PLGA nanoparticles.

Type of NPs	Size ± SD [nm]	Zeta Potential ± SD [mV]	Encapsulation Efficiency ± SD [%]	Drug Loading ± SD [%]
Empty_NPs	214.6 ± 14.0	−9.5 ± 1.0	-	-
GentAOT_NPs	236.7 ± 33.6	−3.7 ± 0.2	99.9 ± 0.1	9.1 ± 0.1

**Table 2:** Composition of the zirconia ceramic suspension.

Ingredients	Mass [g]	Mass Percentage [%]
Basic suspension	180.00	86.26
Distilled water	19.09	9.15
CE 64	0.10	0.05
Glycerine	6.69	3.21
Ethanol	2.78	1.33

## Data Availability

Not applicable.
